# Effect of osteoporotic conditions on the development of peritumoral brain edema after LINAC-based radiation treatment in patients with intracranial meningioma

**DOI:** 10.1186/s13014-021-01890-7

**Published:** 2021-08-23

**Authors:** Sang Mook Kang, Jae Min Kim, Jin Hwan Cheong, Je Il Ryu, Yu Deok Won, Young Soo Kim, Myung-Hoon Han

**Affiliations:** 1 Department of Neurosurgery, Hanyang University Guri Hospital, 153 Gyeongchun-ro, Guri, 471-701 Gyonggi-do Korea; 2 Department of Neurosurgery, Hanyang University Medical Center, 222-1, Wangsimni-ro, Seongdong-gu, Seoul, 133-792 Korea

**Keywords:** Peritumoral brain edema, Meningioma, Osteoporosis, Tumor-brain barrier, Hounsfield unit

## Abstract

**Purpose:**

Disruption of the tumor-brain barrier in meningioma is a crucial factor in peritumoral brain edema (PTBE). We previously reported the possible effect of osteoporosis on the integrity of the arachnoid trabeculae because both the bone and the arachnoid trabeculae are composed of type 1 collagen. We hypothesized that osteoporotic conditions may be associated with PTBE occurrence after radiation treatment in patients with meningioma.

**Methods:**

A receiver operating characteristic curve analysis was used to identify the optimal cut-off values of mean skull Hounsfield unit for predicting osteopenia and osteoporosis in patients from our registry. Multivariate Cox regression analysis was used to determine whether possible osteoporosis independently predicted PTBE development in patients with meningioma after radiation.

**Results:**

A total of 106 intracranial meningiomas were included for the study. All patients received linear accelerator-based radiation therapy in our hospital over an approximate 6-year period. Multivariate Cox regression analysis identified that hypothetical osteoporosis was an independent predictive factor for the development of PTBE in patients with meningioma after linear accelerator-based radiation treatment (hazard ratio 5.20; 95% confidence interval 1.11–24.46; p = 0.037).

**Conclusions:**

Our study suggests that possible osteoporotic conditions may affect PTBE development after linear accelerator-based radiation treatment for intracranial meningioma. However, due to the study’s small number of patients, these findings need to be validated in future studies with larger cohorts, before firm recommendations can be made.

**Graphic abstract:**

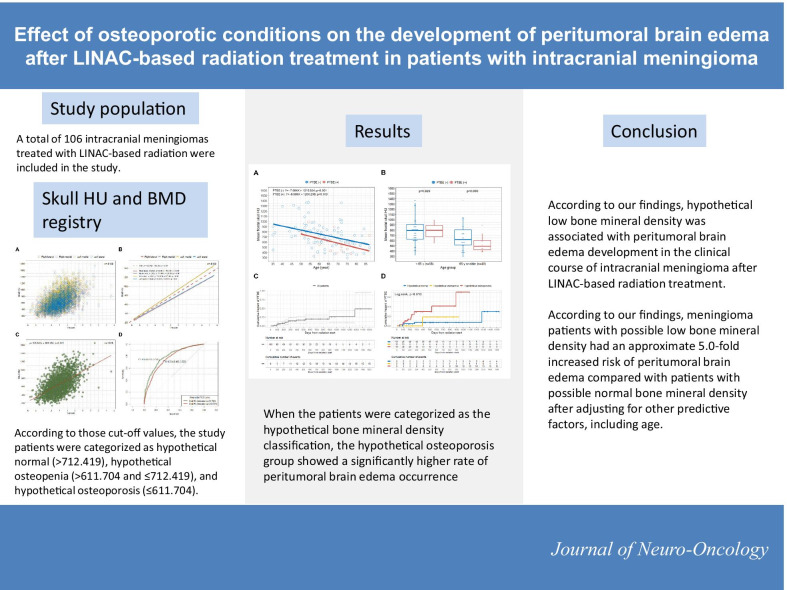

**Supplementary Information:**

The online version contains supplementary material available at 10.1186/s13014-021-01890-7.

## Introduction

Radiation therapy is the primary treatment for patients with small (< 3 cm) asymptomatic tumors or tumors located in the cranial base [1]. While radiation treatment is generally accepted as a safe procedure, symptomatic peritumoral brain edema (PTBE) is the most common complication in intracranial meningioma after radiation therapy and 6–35% of patients experience treatment-related PTBE [2]. Several risk factors such as greater radiation dose, larger tumor size, tumor location, brain-tumor interface, no prior resection for meningioma, atypical histology, and presence of pretreatment edema were reported to be associated with PTBE after radiosurgery in meningioma [2, 3]

Disruption of the tumor-brain barrier in meningioma is an important factor in PTBE development [4]. The brain-meningioma interface is composed of tumor stroma, arachnoid mater, and arachnoid trabeculae [5]. To predict bone mineral density (BMD), we previously reported a method for prediction of osteoporosis by measuring frontal skull Hounsfield unit (HU) values on the brain CT [6]. Both the bone and the arachnoid trabeculae are composed of type 1 collagen. Therefore, we used skull HU values to report the possible effect of systemic osteoporosis on the integrity of the arachnoid trabeculae [6, 7].

Because osteoporotic conditions may negatively affect the integrity of the brain-meningioma interface, which is composed of arachnoid trabeculae, in this study, we examined whether osteoporotic conditions were associated with PTBE occurrence after radiation treatment in patients with meningioma.

## Methods

### Study patients

This study included patients from the NOVALIS registry. The NOVALIS registry was designed for prospective research of patients who received radiation treatment in our hospital [8]. We investigated all consecutive patients with intracranial meningioma from the registry who underwent linear accelerator (LINAC)-based radiation treatment for the first time at our hospital from July 7, 2014 to September 30, 2020.

All meningiomas were diagnosed by radiologic findings alone or pathological confirmation after surgical tumor resection. All radiologic findings were confirmed by experienced neuro-radiologists. We defined PTBE as newly developed PTBE or the progression of preexisting PTBE in follow-up imaging with newly developed neurologic symptoms after radiation treatment [3]. To identify PTBE after radiation treatment, we only included patients with meningioma in the study who met all following conditions: (1) follow-up for at least 6 months, (2) at least one follow-up imaging (CT/magnetic resonance imaging [MRI]), (3) no preexisting PTBE except for patients who underwent surgery for the meningioma before radiation treatment, and (4) measurable intercortical space of the frontal skull on brain CT scan. The last imaging follow-up period after radiation treatment was examined in all patients.

### Radiation treatment

The detailed radiation technique in our hospital was previously described [8]. The NOVALIS Tx system (Varian Medical Systems, CA, USA; Brainlab, Feldkirchen, Germany) was used to treat all meningioma patients. We used noninvasive thermoplastic masks for the simulation-CT for radiation treatment and during the radiation treatment in all patients. To improve the precision of radiation treatment, the Novalis ExacTrac image system and robotic couch of the NOVALIS Tx system were used to adjust the patients’ positions based on the information from the real-time image acquisition.

We used the 3D treatment/planning systems of the NOVALIS Tx, including iPlan (Brainlab, Feldkirchen, Germany) and Eclipse (Varian, CA, USA), for the radiation planning using MRI/CT-fusion images of the patients. The gross tumor volume (GTV), clinical target volume (CTV), and planning target volume (PTV) were automatically calculated by the 3D treatment/planning system of the NOVALIS Tx. We defined the GTV as the exact enhanced area of the meningioma on contrast-enhanced T1-weighted MRI images. For the operated patients, the GTV was defined as the postoperative resection cavity (if available) plus the enhanced area of residual tumor without inclusion of the PTBE area. In patients without surgery and for Grade I benign meningioma, the CTV was identical to the GTV. For Grade II and III meningiomas, the CTV was usually defined as 1–2 cm margin added to the GTV [9]. When the tumor was located near an organ at risk, we reduced the expansion of the CTV margin near the area of the tumor that was close to the organ at risk. The PTV was defined as a symmetrical 0- to 2-mm expansion from the CTV.

We defined the fractionated stereotactic radiotherapy (FSRT) as > 10 sessions (1.8–2.0 Gy/fraction), hypofractionated stereotactic radiotherapy (hFSRT) as 6 to 10 fractions, hypofractionated SRS (hf-SRS) as 2 to 5 fractions, and stereotactic radiosurgery (SRS) as a single session treatment [10, 11]. To compare treatment doses between patients who received radiation with various fractionations, we calculated the biologically effective dose (BED) based on the following equation: BED = nd × (1 + d/3), where n is the fraction, d is the dose of one fraction, and α/β = 3 [12].

### Measurement of frontal skull HU values

We used the simulation-CT images (Philips Brilliance Big Bore CT Simulators) for radiation planning to measure the frontal skull HU values in all patients of the study cohort. We previously described the detailed methods for measuring HU values on frontal cancellous bone on brain CT [6–8, 13]. The frontal skull HU values were measured at each of the four lines on the frontal cancellous bone between the right and left coronal sutures at the CT slice that the lateral ventricles disappear on the brain CT (Additional file 1: Fig. 1). To avoid including cortical bone, all CT images were magnified for the HU value measurement.

### Skull HU and BMD registry

We previously reported the Skull HU and BMD (SHUB) registry in our hospital [6]. In addition to the previous registry (from January 1, 2010 to December 31, 2016), we further enrolled patients (> 18 years old) who had both procedure codes for dual-energy X-ray absorptiometry (DXA) (NMF03) and brain CT (RCG01A and B) in our hospital between January 1, 2017 and December 31, 2019. Following the same protocol as before, the lowest T-score value for patients who underwent multiple DXA scans was used for the analysis. All CT images were obtained using a Siemens CT scanner in our hospital with continuous slices, no gap, and 4.0–5.0-mm slice thickness [6]. When patients received multiple brain CT scans, the brain CT image closest to the date of the selected DXA scan was used. To reduce the time interval heterogeneity, we excluded patients with more than 3 years between DXA and brain CT. Based on the study showing a slow progression to osteoporosis in postmenopausal women, we believe that our within 3-year time interval between the DXA and brain CT scans may be suitable for investigation of the relationship between frontal skull HU values and BMD [14]. In addition, patients with excessively narrow intercortical space of the frontal skull on brain CT were excluded. Therefore, 2025 patients were finally included in the updated SHUB registry.

The BMD was assessed in the lumbar spine (L1–L4) and femoral neck using a Discovery Wi DXA system (Hologic, Bedford, MA, USA) in all patients of the SHUB registry. The lower T-score between the lumbar spine and femoral neck was used as the T-score for the registry. Based on the World Health Organization T-score classification, we defined osteoporosis as a T-score ≤ − 2.5, osteopenia as a T-score > − 2.5 and ≤ − 1.0, and a normal BMD as a T-score > − 1.0.

### Statistical methods

Chi-square and Student’s *t* testing were used to evaluate differences between the PTBE (−) and PTBE (+)groups. We used mean skull HU value ([mean right lateral HU + mean right medial HU + mean left medial HU + mean left lateral HU]/4) in all analysis.

A receiver operating characteristic (ROC) curve analysis was performed to identify the optimal cut-off values of mean skull HU for predicting osteopenia and osteoporosis in the patients of the SHUB registry. Mean frontal skull HU values were used as the test variable, and the individual BMD classification was entered as the state variable (dependent variable) in the ROC curve analysis. When we identified the cut-off skull HU value for predicting osteopenia, we coded the normal BMD (T-score > − 1.0) as 0 and the osteopenia and osteoporosis BMD (T-score ≤ − 1.0) as 1 and input the state variable. In the osteoporosis model, we coded the normal and osteopenia BMD (T-score > − 2.5) as 0 and osteoporosis (T-score ≤ − 2.5) as 1 and input the state variable.

The cumulative hazard for development of PTBE was calculated by Kaplan–Meier analysis classified by hypothetical BMD classification, with censoring of patients who had no PTBE on their last brain CT/MRI after radiation treatment start. Hazard ratios (HRs) with 95% confidence intervals (CIs) were then calculated using a multivariate Cox regression analysis to determine whether the possible osteoporotic condition independently predicted PTBE occurrence in patients with meningioma after radiation treatment. Sex, age (continuous variable), BMI (continuous variable), classification of mean skull HU, location (continuous variable), GTV (continuous variable), BED (continuous variable), fractionation (continuous variable), hypertension, and diabetes were entered into the multivariate model. To balance heterogeneities in patient sex, patient age distribution, tumor location, tumor volume, and radiation dose between the PTBE (−) and PTBE (+) groups, additional propensity score-matched analysis was performed using a multivariable logistic regression model. The model was based on patient sex, patient age, tumor location, GTV, and BED. We matched the PTBE (+) group to the controls in a 1:2 ratio using the greedy nearest-neighbor method using R software [15].

A p value < 0.05 was considered statistically significant. All statistical analyses were performed using R software version 3.6.3 and SPSS for Windows, version 24.0 (IBM, Chicago, IL).

## Results

### Determination of the optimal skull HU values predicting osteopenia and osteoporosis

**Fig. 1:**
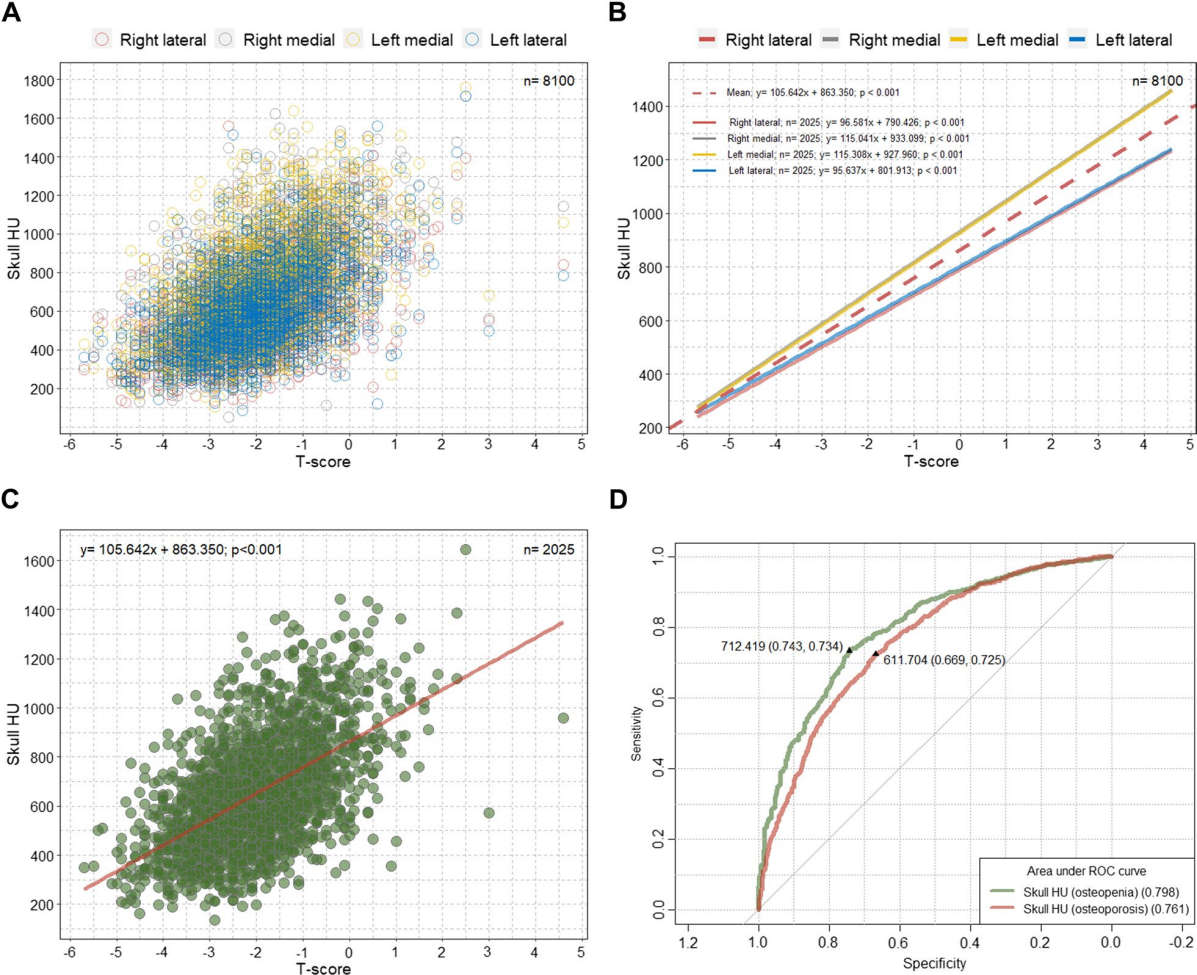
Scatterplot, linear regression line, and ROC curve from the SHUB registry. **A** Overall distribution of HU values at each of four lines according to T-scores; **B** linear regression lines showing the association between the HU value at each of the four lines and the T-score; **C** linear regression line showing the association between the T-score and mean skull HU; **D** ROC curve to determine the optimal cut-off skull HU values for predicting osteopenia and osteoporosis. ROC = receiver operating characteristic; SHUB = skull Hounsfield unit and bone mineral density; HU = Hounsfield unit

There were significant linear associations between T-scores and skull HU values at four different sites on the frontal cancellous bone among the patients in the SHUB registry (Fig. 1A, B). The data in Fig. 1C show an increase of approximately 105.6 mean skull HU value per 1 T-score increase (B = 105.64; p < 0.001). The optimal cut-off value for mean skull HU to predict osteopenia was 712.419 (area under the curve [AUC] = 0.798; p < 0.001), and the HU value to predict osteoporosis was 611.704 (AUC = 0.761; p < 0.001) in the patients from the SHUB registry (Fig. 1D). According to those cut-off values, the study patients were categorized as hypothetical normal (above the cut-off HU value for osteopenia [> 712.419]), hypothetical osteopenia (between the cut-off HU values for osteopenia and osteoporosis [> 611.704 and ≤ 712.419]), and hypothetical osteoporosis (below the cut-off HU value for osteoporosis [≤ 611.704]).

### Characteristics of the study patients

A total of 99 patients with 106 intracranial meningiomas were included for the study. All patients received LINAC-based radiation treatments in our hospital over an approximate 6-year period. A total of 15 patients (14.2%) had PTBE after radiation treatment (Table 1). The mean patient age was 63.3 years and 77.4% of patients were female. A total of 42 patients (39.6%) were categorized as hypothetical osteoporosis. The mean GTV of meningioma and BED were 8.2 cc and 89.5 Gy (Gy), respectively. Patient characteristics are presented in Table 1.

**Table 1:** Characteristics of patients with intracranial meningioma who underwent LINAC-based radiation treatment

Characteristics	PTBE (−)	PTBE (+)	Total	p
Number (%)	91 (85.8)	15 (14.2)	106	
Sex, female, n (%)	70 (76.9)	12 (80.0)	82 (77.4)	0.792
Age, mean ± SD, y	62.4 ± 12.2	69.3 ± 9.3	63.3 ± 12.0	0.039
Imaging follow-up period, median (IQR), days	300.0 (173.0–622.0)	389.0 (243.0–892.0)	319.5 (176.8–635.0)	0.122
BMI, mean ± SD, kg/m^2^	24.5 ± 3.7	24.3 ± 2.9	24.5 ± 3.6	0.861
Classification of mean skull HU, n (%)				0.069
Hypothetical normal (> 712.4)	49 (53.8)	4 (26.7)	53 (50.0)	
Hypothetical osteopenia (> 611.7 and ≤ 712.4)	10 (11.0)	1 (6.7)	11 (10.4)	
Hypothetical osteoporosis (≤ 611.7)	32 (35.2)	10 (66.7)	42 (39.6)	
Prior surgical resection, n (%)	38 (41.8)	4 (26.7)	42 (39.6)	0.268
Pathology, n (%)				0.472
WHO grade I	26 (28.6)	3 (20.0)	29 (27.4)	
WHO grade II	8 (8.8)	0	8 (7.5)	
WHO grade III	4 (4.4)	1 (6.7)	5 (4.7)	
Location, n (%)				0.730
Convexity	30 (33.0)	6 (40.0)	36 (34.0)	
Parasagittal or parafalcine	19 (20.9)	5 (33.3)	24 (22.6)	
Sphenoid ridge	9 (9.9)	1 (6.7)	10 (9.4)	
Cerebellopontine angle	11 (12.1)	2 (13.3)	13 (12.3)	
Posterior fossa	8 (8.8)	1 (6.7)	9 (8.5)	
Parasellar or petroclival	11 (12.1)	0	11 (10.4)	
Other	3 (3.3)	0	3 (2.8)	
GTV, mean ± SD, cc	7.6 ± 9.5	11.8 ± 9.4	8.2 ± 9.6	0.118
PTV, mean ± SD, cc	10.8 ± 12.7	16.4 ± 10.8	11.6 ± 12.6	0.110
Marginal radiation dose, mean ± SD, Gy	30.9 ± 11.4	27.5 ± 5.6	30.4 ± 10.8	0.262
Fractionation, n (%)				0.360
SRS	16 (17.6)	3 (20.0)	19 (17.9)	
hf-SRS (2–5 fractions)	51 (56.0)	10 (66.7)	61 (57.5)	
hFSRT (6–10 fractions)	8 (8.8)	2 (13.3)	10 (9.4)	
FSRT	16 (17.6)	0	16 (15.1)	
Dose per fraction, mean ± SD, Gy	7.0 ± 5.1	8.0 ± 5.2	7.1 ± 5.1	0.492
BED (α/β = 3), mean ± SD, Gy	89.0 ± 18.4	92.6 ± 21.0	89.5 ± 18.7	0.497
Past medical history, n (%)				
Hypertension	38 (41.8)	7 (46.7)	45 (42.5)	0.722
Diabetes	14 (15.4)	3 (20.0)	17 (16.0)	0.652

LINAC, linear accelerator; PTBE, peritumoral brain edema; SD, standard deviation; IQR, interquartile range; BMI, body mass index; HU, Hounsfield unit; WHO, world health organization; GTV, gross tumor volume; PTV, planning target volume; Gy, gray; SRS, stereotactic radiosurgery; hf-SRS, hypofractionated stereotactic radiosurgery; hFSRT, hypofractionated stereotactic radiotherapy; FSRT, fractionated stereotactic radiotherapy; BED, biologically equivalent dose

### Skull HU values based on PTBE in the study cohort and HU values and BMD in the SHUB registry cohort

Detailed information about the skull HU values according to PTBE in the study cohort and the skull HU values with additional BMD information for the SHUB registry patients is presented in Table 2. The SHUB registry showed relatively a high proportion of women and patients with an older age distribution than the study patients. The overall average mean frontal skull HU value was 716.2 in the study patients and 653.0 among the SHUB registry patients. In the study cohort, there were significant differences in skull HU values between the PTBE (−) and PTBE (+) groups. The median time between brain CT and BMD measurement was 151 days, and 36.6% of patients in the SHUB registry were diagnosed with osteoporosis.

**Table 2:** Descriptive information of skull HU values according to the development of PTBE in the study and skull HU values and BMD in the SHUB registry cohorts

Variables	Study cohort				SHUB registry
	PTBE (−)	PTBE (+)	Total	p	
Number	91	15	106		2025
Sex				0.792	
Female, n (%)	70 (76.9)	12 (80.0)	82 (77.4)		1704 (84.1)
Age, median (IQR), y	61.0 (54.0–72.0)	72.0 (66.0–75.0)	62.5 (54.8–72.0)	0.039	69.0 (59.0–77.0)
Age, mean ± SD, y	62.4 ± 12.2	69.3 ± 9.3	63.3 ± 12.0	0.039	67.9 ± 11.9
Overall mean skull HU value, median (IQR)	730.8 (581.5–874.0)	547.8 (430.5–725.8)	711.6 (547.2–860.7)	0.025	625.7 (483.8–791.5)
Overall mean skull HU value, mean ± SD	737.9 ± 248.9	584.5 ± 189.4	716.2 ± 246.6	0.025	653.0 ± 229.9
Mean HU value at each of four sites in the frontal skull, mean ± SD					
Right lateral	700.3 ± 238.3	582.5 ± 133.7	683.6 ± 229.7	0.065	598.1 ± 220.4
Right medial	780.8 ± 287.3	615.5 ± 229.1	757.4 ± 284.8	0.037	704.1 ± 263.6
Left medial	754.5 ± 283.1	599.5 ± 250.0	732.6 ± 282.8	0.049	698.4 ± 262.4
Left lateral	716.2 ± 255.2	540.7 ± 178.8	691.3 ± 252.7	0.012	611.5 ± 224.8
Average, medial	767.7 ± 281.5	607.5 ± 235.2	745.0 ± 280.1	0.040	701.2 ± 257.9
Average, lateral	708.2 ± 240.1	561.6 ± 153.6	687.5 ± 234.9	0.024	604.8 ± 216.5
Time interval between brain CT and BMD, median (IQR), days	N/A	N/A	N/A		151.0 (9.0–487.0)
T-score, mean ± SD	N/A	N/A	N/A		− 1.99 ± 1.22
Lumbar spine	N/A	N/A	N/A		− 1.65 ± 1.43
Femur neck	N/A	N/A	N/A		− 1.40 ± 1.20
BMD categories, n (%)					
Normal (T-score > − 1.0)	N/A	N/A	N/A		381 (18.8)
Osteopenia (T-score > − 2.5 and ≤ 1.0)	N/A	N/A	N/A		902 (44.5)
Osteoporosis (T-score ≤ -2.5)	N/A	N/A	N/A		742 (36.6)

HU, Hounsfield unit; PTBE, peritumoral brain edema; BMD, bone mineral density; SHUB, skull Hounsfield unit and bone mineral density; IQR, interquartile range; SD, standard deviation; CT, computed tomography; N/A, not available

### Association between age and skull HU according to the PTBE

**Fig. 2:**
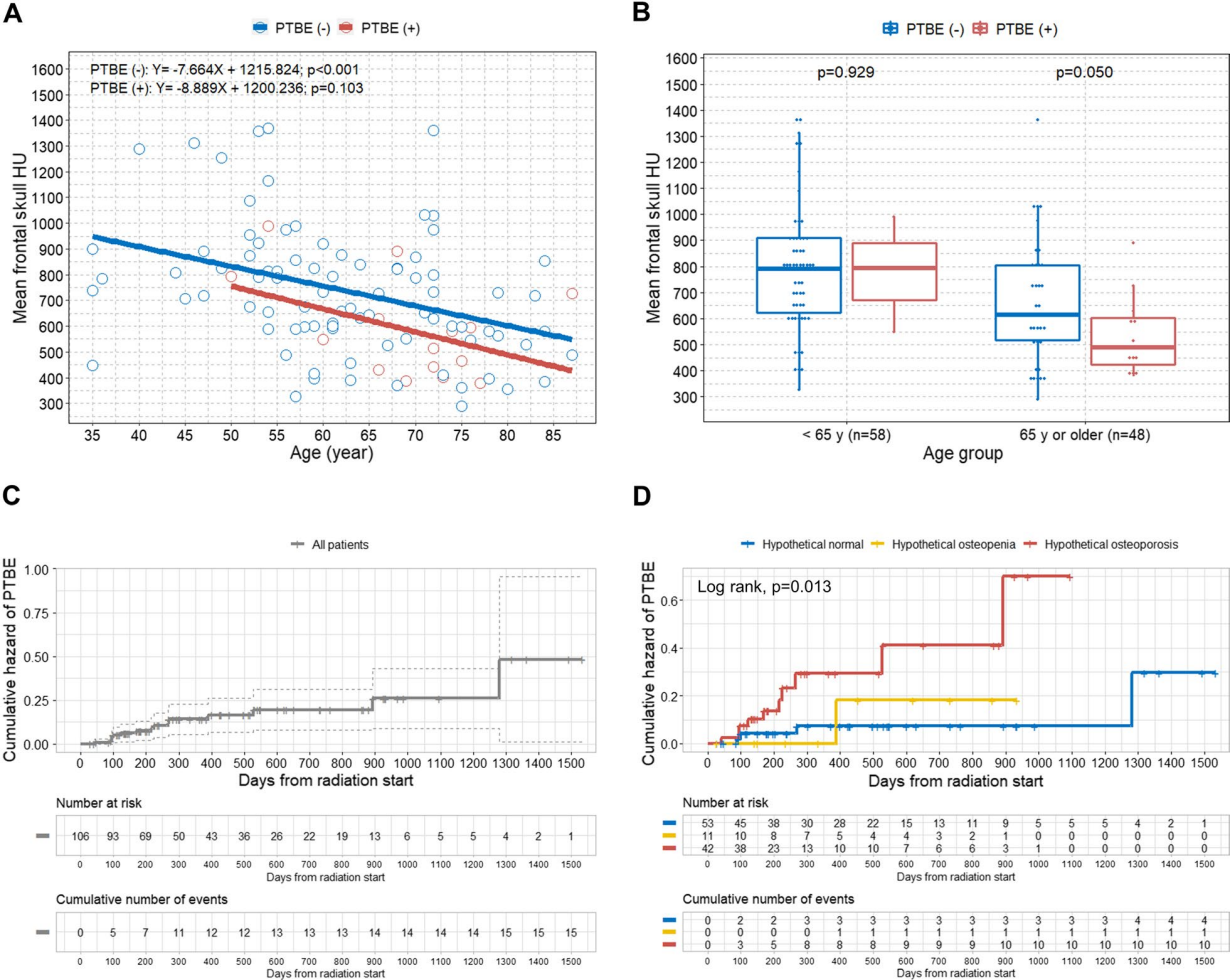
Scatterplot with linear regression lines, boxplots, and Kaplan–Meier curves in the study patients. **A** Scatterplot with linear regression lines showing the association between age and mean frontal skull HU values based on the presence of PTBE; **B** boxplots with dot plots of the mean frontal skull HU values classified by age group according to the presence of PTBE; **C** overall cumulative hazard of PTBE development; **D** cumulative hazard of PTBE development according to the hypothetical BMD groups. HU = Hounsfield unit; PTBE = peritumoral brain edema; BMD = bone mineral density

We observed a significant negative correlation between age and mean skull HU value in the PTBE (−) group (p < 0.001). Although not statistically significant, a similar tendency of a negative association was also observed in the PTBE (+) group (p = 0.103) (Fig. 2A). We found that the PTBE (+) group showed relatively lower mean skull HU values across the age ranges compared with the PTBE (−) group. The PTBE (+) group showed marginally significant lower mean skull HU values compared with the PTBE (−) group among the older age group (p = 0.050) (Fig. 2B).

### Association between possible osteoporosis and PTBE in meningioma after radiation

The overall cumulative hazard for PTBE development in patients with intracranial meningioma after radiation treatment is shown in Fig. 2C. When the patients were categorized as the hypothetical BMD classification, the hypothetical osteoporosis group showed a significantly higher rate of PTBE occurrence (p = 0.013) (Fig. 2D). Multivariate Cox regression analysis determined that hypothetical osteoporosis was an independent predictor for the development of PTBE in patients with meningioma after LINAC-based radiation treatment (HR 5.20; 95% CI 1.11–24.46; p = 0.037) (Table 3). The additional independent predictive factors of PTBE were older age and larger GTV.

**Table 3:** Uni- and multivariate Cox regression analyses for the development of PTBE in patients with intracranial meningioma after LINAC-based radiation treatment based on various predictors

	Univariate analysis		Multivariate analysis	
Variable	HR (95% CI)	p	HR (95% CI)	p
Sex				
Male	Reference		Reference	
Female	1.08 (0.30–3.88)	0.905	0.64 (0.15–2.71)	0.543
Age (per 1 year increase)	1.07 (1.02–1.13)	0.005	1.08 (1.01–1.15)	0.032
BMI (per 1 BMI increase)	0.97 (0.82–1.14)	0.722	1.09 (0.88–1.35)	0.419
Classification of mean skull HU				
Hypothetical normal	Reference		Reference	
Hypothetical osteopenia	1.67 (0.17–16.13)	0.657	3.22 (0.23–45.58)	0.387
Hypothetical osteoporosis	5.45 (1.48–20.03)	0.011	5.20 (1.11–24.46)	0.037
Location	0.70 (0.49–1.00)	0.050	0.70 (0.46–1.06)	0.094
GTV (per 1 cc increase)	1.08 (1.02–1.15)	0.010	1.12 (1.04–1.21)	0.004
BED (α/β = 3) (per 1 Gy increase)	1.01 (0.98–1.03)	0.665	1.02 (0.97–1.06)	0.484
Fractionation (per 1 fraction increase)	0.94 (0.85–1.03)	0.179	0.90 (0.71–1.16)	0.415
Hypertension				
No	Reference		Reference	
Yes	1.13 (0.41–3.15)	0.815	0.66 (0.17–2.61)	0.554
Diabetes				
No	Reference		Reference	
Yes	1.05 (0.29–3.79)	0.937	0.42 (0.09–2.03)	0.278

PTBE, peritumoral brain edema; HR, hazard ratio; CI, confidence interval; BMI, body mass index; HU, Hounsfield unit; GTV, gross tumor volume; BED, biologically equivalent dose; Gy, gray

We also performed additional propensity score-matched analyses based on patient sex, patient age, tumor location, GTV, and BED (Table 4). We found that hypothetical osteoporosis remained an independent predictive factor for the development of PTBE after LINAC-based radiation treatment in the propensity score-matched patients (HR 5.36; 95% CI 1.06–27.12; p = 0.042) (Table 5).

**Table 4:** Characteristics of patients with intracranial meningioma who underwent LINAC-based radiation treatment according to the presence of PTBE before and after propensity score matching based on patient sex, patient age, tumor location, GTV, and BED

Characteristics	Before propensity score matching			After propensity score matching		
	PTBE (−) (n = 91)	PTBE (+) (n = 15)	p	PTBE (−) (n = 30)	PTBE (+) (n = 15)	p
Sex, female, n (%)	70 (76.9)	12 (80.0)	0.792	23 (76.7)	12 (80.0)	0.800
Age, mean ± SD, y	62.4 ± 12.2	69.3 ± 9.3	0.039	68.2 ± 11.0	69.3 ± 9.3	0.741
Location, n (%)			0.730			0.777
Convexity	30 (33.0)	6 (40.0)		13 (43.3)	6 (40.0)	
Parasagittal or parafalcine	19 (20.9)	5 (33.3)		10 (33.3)	5 (33.3)	
Sphenoid ridge	9 (9.9)	1 (6.7)		3 (10.0)	1 (6.7)	
Cerebellopontine angle	11 (12.1)	2 (13.3)		1 (3.3)	2 (13.3)	
Posterior fossa	8 (8.8)	1 (6.7)		3 (10.0)	1 (6.7)	
Parasellar or petroclival	11 (12.1)	0		0	0	
Other	3 (3.3)	0		0	0	
GTV, mean ± SD, cc	7.6 ± 9.5	11.8 ± 9.4	0.118	9.5 ± 12.2	11.8 ± 9.4	0.523
BED (α/β = 3), mean ± SD, Gy	89.0 ± 18.4	92.6 ± 21.0	0.497	93.3 ± 19.9	92.6 ± 21.0	0.905
Classification of mean skull HU, n (%)			0.069			0.447
Hypothetical normal (> 712.4)	49 (53.8)	4 (26.7)		13 (43.3)	4 (26.7)	
Hypothetical osteopenia > 611.7 and ≤ 712.4)	10 (11.0)	1 (6.7)		3 (10.0)	1 (6.7)	
Hypothetical osteoporosis (≤ 611.7)	32 (35.2)	10 (66.7)		14 (46.7)	10 (66.7)	

LINAC, linear accelerator; PTBE, peritumoral brain edema; GTV, gross tumor volume; BED, biologically equivalent dose; SD, standard deviation; Gy, gray; HU, Hounsfield unit

**Table 5:** Univariate and multivariate Cox regression analyses of PTBE development in patients with intracranial meningioma after LINAC-based radiation treatment after propensity score matching based on patient sex, patient age, tumor location, GTV, and BED

	Univariate analysis		Multivariate analysis	
Variable	HR (95% CI)	p	HR (95% CI)	p
Sex				
Male	Reference		Reference	
Female	0.83 (0.23–2.98)	0.774	0.44 (0.08–2.37)	0.339
Age (per 1 year increase)	1.03 (0.97–1.08)	0.357	1.02 (0.95–1.09)	0.671
Location	0.98 (0.67–1.45)	0.932	1.09 (0.67–1.78)	0.732
GTV (per 1 cc increase)	1.05 (0.99–1.11)	0.095	1.09 (1.01–1.17)	0.028
BED (α/β = 3) (per 1 Gy increase)	0.99 (0.96–1.02)	0.592	1.02 (0.99–1.06)	0.210
Classification of mean skull HU				
Hypothetical normal	Reference		Reference	
Hypothetical osteopenia	2.90 (0.29–29.30)	0.368	5.67 (0.40–81.37)	0.202
Hypothetical osteoporosis	3.88 (1.02–14.81)	0.047	5.36 (1.06–27.12)	0.042

PTBE, peritumoral brain edema; LINAC, linear accelerator; GTV, gross tumor volume; BED, biologically equivalent dose; HR, hazard ratio; CI, confidence interval; Gy, gray; HU, Hounsfield unit

## Discussion

We found that hypothetical low BMD was associated with PTBE development in the clinical course of intracranial meningioma after LINAC-based radiation treatment. According to our findings, meningioma patients with possible low BMD had an approximate 5.0-fold increased risk of PTBE compared with patients with possible normal BMD after adjusting for other predictive factors, including age. Because PTBE development in meningioma after radiation is associated with meningioma location, meningioma volume, and radiation dose [3], we further needed to reduce the confounding effects of these risk factors on the true association between osteoporotic conditions and PTBE occurrence. Therefore, although we had adjusted for these risk factors in the multivariate analysis, we performed an additional propensity score-matched analysis, which showed that hypothetical osteoporosis remained an independent predictor of PTBE development in patients with meningioma after radiation treatment.

The HU values in a specific anatomical area on a CT image and BMD T-scores are absolute values [16, 17]. Therefore, the correlation between the HU value and real T-score might not be influenced by the different characteristics of patients between the study cohort and SHUB registry. This is because patient characteristics can affect bone quality, but they might not influence the simple correlation between the absolute values of HU and BMD [6]. A previous study also determined optimal lumbar spine HU values for predicting osteoporosis from abdominal CT images obtained from heterogeneous patients for other reasons [18]. Other studies also reported that cancellous bone HU values in various anatomical areas on CT scans are strongly associated with T-score and may be useful in detecting osteoporosis [19–21]. We previously showed that the frontal skull HU value also can predict osteoporotic conditions [6].

Tumor-brain barrier disruption is a crucial component of PTBE formation in meningioma [4]. Previous studies examining the brain-meningioma interface showed that the degree of arachnoid disruption was associated with the progression of brain edema [5]. The arachnoid membrane acts as mechanical and biochemical buffer against the spread of edema-associated proteins and vasogenic edema fluids from meningiomas [1, 2].

A previous study that examined the microscopic anatomy of the brain-meningioma interface reported that the brain-meningioma interface is composed of tumor stroma, arachnoid mater, and arachnoid trabeculae [5]. The arachnoid mater is composed of two layers and an inner part is the arachnoid trabeculae supporting the stability of the subarachnoid space [22]. Meningioma originates from the arachnoid cap cell [23]. Therefore, it is naturally hypothesized that meningioma originated from arachnoid cap cells may push the arachnoid trabeculae, which is inner part of arachnoid membrane, into the pia mater [24]. As the tumor grows, arachnoid trabeculae may be sandwiched between the pia mater and meningioma, and this may form the brain-meningioma interface.

Type 1 collagen is a major component of bone, and its gene mutation causes osteoporosis [25, 26]. Interestingly, the arachnoid trabeculae is also composed of type 1 collagen [27]. Because osteoporosis is a systemic disease that affects systemic BMD and microarchitecture throughout the body, it is reasonable to postulate that osteoporosis may also influence the arachnoid trabeculae, which is also composed of type 1 collagen [28]. We recently showed the possible association between systemic osteoporosis and the structural integrity of arachnoid trabeculae based on the above concept that both the bone and arachnoid trabeculae are composed of the same type 1 collagen [6, 7]. Supporting our hypothesis, osteogenesis imperfecta caused by mutations in type 1 collagen genes (*COL1A1*/*COL1A2*) is associated with communicating hydrocephalus [29]. Therefore, we hypothesized that arachnoid trabeculae in the brain-meningioma interface may be more damaged as tumors grow in patients with osteoporotic conditions. In addition, radiation affects collagen structure and can cause collagen changes and damage [30–32]. Therefore, when the meningioma is treated with radiation, damage may be increased in the brain-meningioma interface, including the arachnoid trabeculae, in addition to the own effect of the tumor size on this contact interface [1].

Based on the above assumptions, we speculate that a larger tumor would be associated with a greater the likelihood of damage to the brain-meningioma contact interface. This damage to the contact interface may be more severe in osteoporotic patients, as the arachnoid trabeculae, a component of the brain-meningioma interface, may be weakened in osteoporotic conditions since both bone and arachnoid trabeculae are composed of the same type 1 collagen. Radiation therapy may aggravate the damaged brain-meningioma interface and may lead to tumor-brain barrier disruption. Therefore, more weakened the arachnoid trabeculae in the brain-meningioma interface due to osteoporotic conditions may have a higher possibility of having the contact interface disruption after radiation treatment.

Meningioma size is a predictive factor for PTBE in meningioma after radiation treatment [1, 2]. We observed that elderly patients more frequently showed PTBE than younger patients. Because osteoporosis is more common with increasing age, we speculate that elderly patients may have a greater chance of having weakened brain-meningioma interface integrity. In addition, volumetric loss in cerebral white matter and loosening of the microstructure network may lead to direct transmission of edematous fluids into the white matter and increase the possibility of PTBE [33]. We also found that BED was not associated with PTBE development. We believe that this is because we rarely used extremely high radiation doses, and a narrowed dose range may not cause significant differences in PTBE development [1].

Our study has some limitations. First, due to its retrospective nature, the study has inherent limitations. Second, the small sample size of the study may have low power, and our findings need to be confirmed by further studies. Third, heterogeneity in tumor location and absence of pathological confirmation in many cases may bias the results. Fourth, there were some differences in known risk factors for PTBE occurrence, including patient age, tumor location and volume, and radiation dose, in meningioma after radiation between the PTBE (−) and PTBE (+) groups. Therefore, although these risk factors were adjusted in multivariate analysis, we additionally performed propensity score-matched analysis to further reduce the confounding effects of these risk factors on the true association between osteoporotic conditions and PTBE occurrence. Finally, although skull HU value showed a strong correlation with BMD, this may not reflect the actual T-score.

In conclusion, our study proposes that possible osteoporotic conditions may affect PTBE development after LINAC-based radiation treatment for intracranial meningioma. Clinical brain CT scans may allow the detection of possible osteoporosis by using a convenient method to measure HU in the frontal skull on brain CT. However, due to the study’s small sample size, these findings need to be confirmed in future studies with larger cohorts for the recommend of caution regarding PTBE development in osteoporotic patients after radiation.

## Supplementary Information

**Additional file 1. Fig. 1.** Measurement of HU values at each of four lines on the frontal bone. The PACS automatically calculated the maximum, minimum, and mean HU values according to the values on the drawn line. The mean HU value on each of the four lines was recorded. HU=Hounsfield unit; PACS=picture archiving and communication system.

## Data Availability

The datasets used and/or analyzed during the current study are available from the corresponding author on reasonable request.

## Abbreviations

PTBE: Peritumoral brain edema
BMD: Bone mineral density
HU: Hounsfield unit
LINAC: Linear accelerator
MRI: Magnetic resonance imaging
GTV: Gross tumor volume
CTV: Clinical target volume
PTV: Planning target volume
FSRT: Fractionated stereotactic radiotherapy
hFSRT: Hypofractionated stereotactic radiotherapy
hf-SRS: Hypofractionated stereotactic radiosurgery
BED: Biologically effective dose
ROC: Receiver operating characteristic
HR: Hazard ratio
CI: Confidence interval

## References

[1] Cai R, Barnett GH, Novak E (2010). Principal risk of peritumoral edema after stereotactic radiosurgery for intracranial meningioma is tumor-brain contact interface area. Neurosurgery.

[2] Conti A, Pontoriero A, Siddi F (2016). Post-treatment edema after meningioma radiosurgery is a predictable complication. Cureus.

[3] Milano MT, Sharma M, Soltys SG (2018). Radiation-induced edema after single-fraction or multifraction stereotactic radiosurgery for meningioma: a critical review. Int J Radiat Oncol.

[4] Hou J, Kshettry VR, Selman WR, Bambakidis NC (2013). Peritumoral brain edema in intracranial meningiomas: the emergence of vascular endothelial growth factor-directed therapy. Neurosurg Focus.

[5] Nakasu S, Fukami T, Jito J, Matsuda M (2005). Microscopic anatomy of the brain-meningioma interface. Brain Tumor Pathol.

[6] Han M-H, Won YD, Na MK (2018). Association between possible osteoporosis and shunt-dependent hydrocephalus after subarachnoid hemorrhage. Stroke.

[7] Deok WY, Min KJ, Hwan CJ (2021). Effect of osteoporotic condition on ventriculomegaly and shunt-dependent hydrocephalus after subarachnoid hemorrhage. Stroke.

[8] Lee R-H, Kim JM, Cheong JH (2020). Significance of skull osteoporosis to the development of peritumoral brain edema after LINAC-based radiation treatment in patients with intracranial meningioma. PLoS ONE.

[9] Combs SE, Baumert BG, Bendszus M (2021). ESTRO ACROP guideline for target volume delineation of skull base tumors. Radiother Oncol J Eur Soc Ther Radiol Oncol.

[10] Meniai-Merzouki F, Bernier-Chastagner V, Geffrelot J (2018). Hypofractionated stereotactic radiotherapy for patients with intracranial meningiomas: impact of radiotherapy regimen on local control. Sci Rep.

[11] Kirkpatrick JP, Soltys SG, Lo SS (2017). The radiosurgery fractionation quandary: single fraction or hypofractionation?. Neuro-Oncol.

[12] Patil CG, Hoang S, Borchers DJ (2008). Predictors of peritumoral edema after stereotactic radiosurgery of supratentorial meningiomas. Neurosurgery.

[13] Won YD, Na MK, Kim CH (2018). The frontal skull Hounsfield unit value can predict ventricular enlargement in patients with subarachnoid haemorrhage. Sci Rep.

[14] Gourlay ML, Fine JP, Preisser JS (2012). Bone-density testing interval and transition to osteoporosis in older women. N Engl J Med.

[15] Randolph J, Falbe K, Manuel A, Balloun J (2019). A step-by-step guide to propensity score matching in R. Pract Assess Res Eval.

[16] Birnbaum BA, Hindman N, Lee J, Babb JS (2007). Multi-detector row CT attenuation measurements: assessment of intra- and interscanner variability with an anthropomorphic body CT phantom. Radiology.

[17] Klippel JH, Stone JH, Crofford LJ, White PH (2008). Primer on the rheumatic diseases.

[18] Pickhardt PJ, Pooler BD, Lauder T (2013). Opportunistic screening for osteoporosis using abdominal computed tomography scans obtained for other indications. Ann Intern Med.

[19] Choi MK, Kim SM, Lim JK (2016). Diagnostic efficacy of Hounsfield units in spine CT for the assessment of real bone mineral density of degenerative spine: correlation study between T-scores determined by DEXA scan and Hounsfield units from CT. Acta Neurochir (Wien).

[20] Zou D, Li W, Deng C (2019). The use of CT Hounsfield unit values to identify the undiagnosed spinal osteoporosis in patients with lumbar degenerative diseases. Eur Spine J.

[21] Johnson CC, Gausden EB, Weiland AJ (2016). Using Hounsfield units to assess osteoporotic status on wrist computed tomography scans: comparison with dual energy X-ray absorptiometry. J Hand Surg.

[22] Yamashima T, Lee JH (2009). Human meninges: anatomy and its role in meningioma pathogenesis. Meningiomas.

[23] Wiemels J, Wrensch M, Claus EB (2010). Epidemiology and etiology of meningioma. J Neurooncol.

[24] Themes UFO. 3 anatomy and biology of the leptomeninges. In: Neupsy Key. https://neupsykey.com/3-anatomy-and-biology-of-the-leptomeninges/ (2020). Accessed 30 Apr 2021

[25] Grant SF, Reid DM, Blake G (1996). Reduced bone density and osteoporosis associated with a polymorphic Sp1 binding site in the collagen type I alpha 1 gene. Nat Genet.

[26] Gajko-Galicka A (2002). Mutations in type I collagen genes resulting in osteogenesis imperfecta in humans. Acta Biochim Pol.

[27] Saboori P, Sadegh A (2015). Histology and morphology of the brain subarachnoid trabeculae. Anat Res Int.

[28] Brandi ML (2009). Microarchitecture, the key to bone quality. Rheumatol Oxf Engl.

[29] Charnas LR, Marini JC (1993). Communicating hydrocephalus, basilar invagination, and other neurologic features in osteogenesis imperfecta. Neurology.

[30] Keskikuru R, Jukkola A, Nuutinen J (2004). Radiation-induced changes in skin type I and III collagen synthesis during and after conventionally fractionated radiotherapy. Radiother Oncol J Eur Soc Ther Radiol Oncol.

[31] Maslennikova A, Kochueva M, Ignatieva N (2015). Effects of gamma irradiation on collagen damage and remodeling. Int J Radiat Biol.

[32] Miller JP, Borde BH, Bordeleau F (2018). Clinical doses of radiation reduce collagen matrix stiffness. APL Bioeng.

[33] Gunning-Dixon FM, Brickman AM, Cheng JC, Alexopoulos GS (2009). Aging of cerebral white matter: a review of MRI findings. Int J Geriatr Psychiatry.

