# Supplementary material for: Effect of osteoporotic conditions on the development of peritumoral brain edema after LINAC-based radiation treatment in patients with intracranial meningioma
Source: Radiat Oncol. 2021 Aug 23;16:160. doi: 10.1186/s13014-021-01890-7 (PMC8383364; doi:10.1186/s13014-021-01890-7)
Supplement: Supplementary file 1 — Additional file 1. Fig. 1. Measurement of HU values at each of four lines on the frontal bone. The PACS automatically calculated the maximum, minimum, and mean HU values according to the values on the drawn line. The mean HU value on each of the four lines was recorded. HU=Hounsfield unit; PACS=picture archiving and communication system. [file 13014_2021_1890_MOESM1_ESM.docx]

**Electronic supplementary material**

**Effect of osteoporotic conditions on the development of peritumoral brain edema after LINAC-based radiation treatment in patients with intracranial meningioma**

**Journal of Neuro-Oncology**

Authors: Sang Mook Kang, M.D., Jae Min Kim, M.D., Ph.D., Jin Hwan Cheong, M.D., Ph.D., Je Il Ryu, M.D., Ph.D., Yu Deok Won, M.D., Young Soo Kim, M.D., Ph.D., Myung-Hoon Han, M.D., Ph.D.

***Corresponding author:** Myung-Hoon Han, M.D., Ph.D.

Department of Neurosurgery, Hanyang University Guri Hospital, 153 Gyeongchun-ro, Guri 471-701, Gyonggi-do, Korea

Tel.: +82.31-560-2328

Fax: +82.31-560-2327

E-mail: gksmh80@gmail.com

ORCID ID: 0000-0003-1728-5017


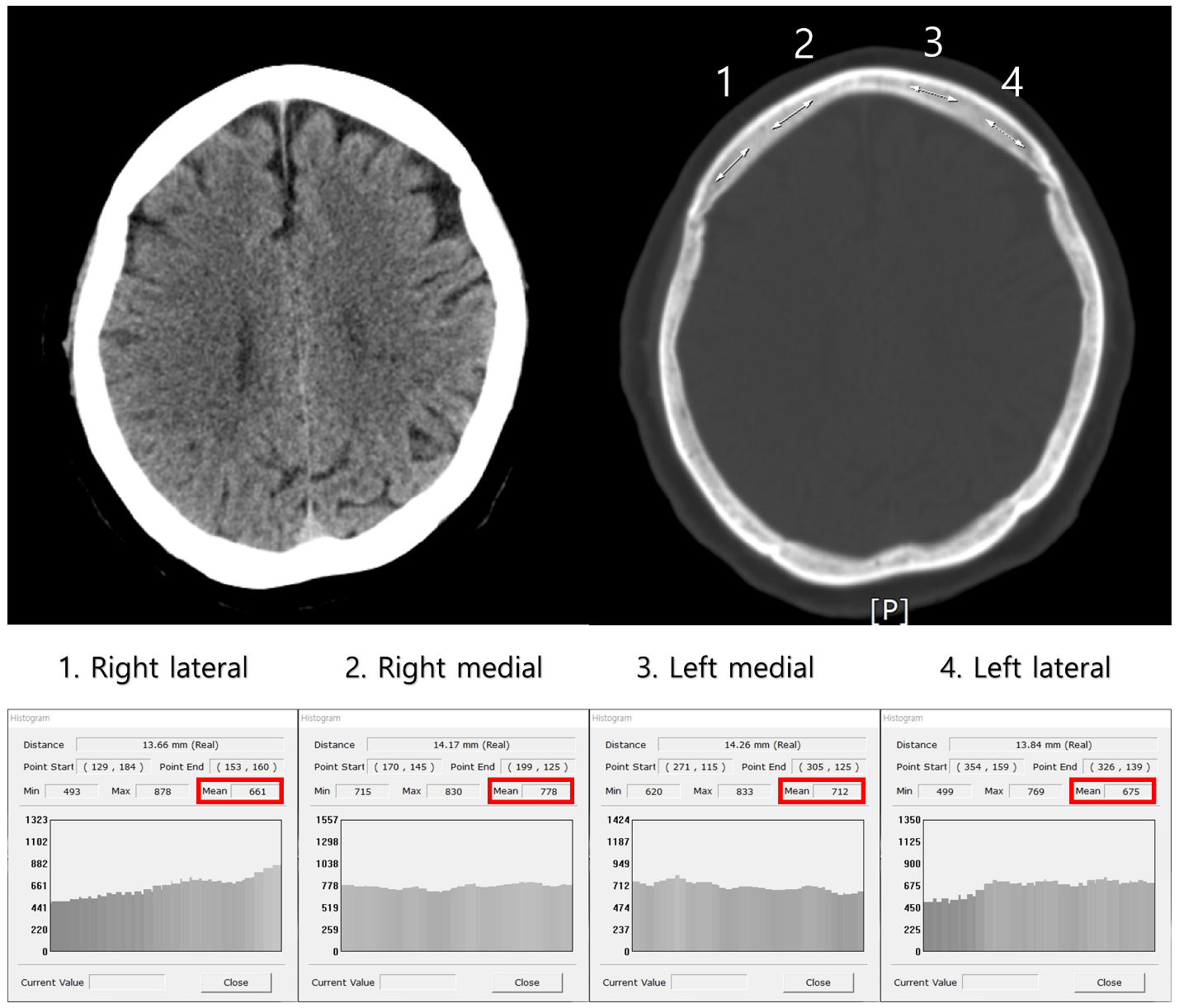


**Suppl. Fig. 1** Measurement of HU values at each of four lines on the frontal bone

The PACS automatically calculated the maximum, minimum, and mean HU values according to the values on the drawn line. The mean HU value on each of the four lines was recorded. HU=Hounsfield unit; PACS=picture archiving and communication system.
